# Dealing With Radicalised Youth Offenders: The Development and Implementation of a Youth-Specific Framework

**DOI:** 10.3389/fpsyt.2021.773545

**Published:** 2022-01-13

**Authors:** Steven Barracosa, James March

**Affiliations:** Youth Justice New South Wales, Department of Communities and Justice, Sydney, NSW, Australia

**Keywords:** youth radicalisation, at-risk youth, countering violent extremism, diversion and disengagement, youth terrorism offenders, youth offenders

## Abstract

**Background:** In 2018 in the Australian State of New South Wales, a specialist Countering Violent Extremism Unit was established in the youth criminal justice system. This was in direct response to a number of youth below the age of 18 who have been charged for terrorism offences and identified as involved in violent extremist acts. This youth-specific framework was the first of its kind in Australia. It was designed to provide multidisciplinary practitioner-based approaches for the early-identification, diversion, and disengagement of at-risk and radicalised youth offenders.

**Aims:** This paper will explore the experiences and lessons learned by the Youth Justice New South Wales Countering Violent Extremism Unit. It will discuss the relevance of youth radicalisation within Australia's evolving national security climate. This includes emerging trends in relation to youth radicalisation to varied violent extremist ideologies. This paper will explore the specialist approach adopted for preventing and countering violent extremism through the identification, assessment, and case management of at-risk and radicalised youth offenders.

**Implications:** The Youth Justice New South Wales experience indicates that youth criminal justice settings can be designed to tackle the challenges posed by at-risk and radicalised youth. The practitioner experience canvassed in this paper highlights that a pluralistic and non-punitive approach to supervision, client-focused assessment and case management processes, and widespread resourcing of multidisciplinary practitioners and programs can be used to account for developmental and psychosocial vulnerabilities in addition to violent extremism risk factors amongst youth offenders. These approaches should be supplemented by youth-specific countering violent extremism practitioner expertise, and a range of violent extremism case management and risk assessment measures.

## Introduction

Radicalised youth have emerged as a “growing threat for the international community” [(1), p. 412]. For example, the Federal Bureau of Investigation reported that American “youth are embracing many forms of violent extremism” [(2), p. 3]. Comparable trends have been identified in the United Kingdom and Europe (3–6). Radicalised youth have been implicated in Western terrorism plots (4, 6, 7). They are part of a demographic that have joined international conflicts in increasing numbers (8). Radicalised youth are now disproportionately represented across violent extremist networks worldwide (9–11).

In Australia, similar trends have been observed. Over 10% of domestic terrorism convictions were for youth below the age of 18 during the period 2014–2018 (12). All of these convictions related to youth inspired by Sunni-Islamist extremism. A further 25% were aged between 18 and 25 (12). Open source studies indicate that Australian youth radicalised to violent extremism are influenced by factors including social dislocation, peer influence, active engagement with extremism online, and triggering events (13, 14). An increase in the presence of Australian youth deemed at-risk of radicalisation has also been identified (13, 15). Researchers content that this relates to vulnerable youth displaying signs of radicalisation before establishing a commitment to engage in ideologically motivated violence (16, 17). Australian youth are now being radicalised to varied violent extremist ideologies (18, 19). It is subsequently argued that the risks posed to national security by radicalised Australian youth is a significant issue that will continue (12).

The presence of radicalised Australian youth has emerged in-line with an increase in domestic approaches to prevent and counter violent extremism. The Australian Government has supported the implementation of a variety of programs and initiatives. These have been designed to supplement law enforcement, security, and intelligence measures (20, 21). This has included approaches to assess and intervene with at-risk and radicalised youth (15, 22, 23). It is argued that the process of youth radicalisation differs from adults due to risk factors and vulnerabilities that are unique to childhood and adolescence (14, 24–26). It is subsequently recommended that countering violent extremism (CVE) approaches should be individually-tailored to address the impact of youth-specific life-course experiences, trajectories, and developmental vulnerabilities on the process of radicalisation (15, 22, 27, 28). It is noted that the Australian Government views radicalisation as a process where people's thinking and behaviour moves outside the mainstream that may result in the use of ideologically motivated violence (https://www.livingsafetogether.gov.au/resources). This is in-line with academic recommendations for radicalisation to be viewed as both a non-violent and violent construct [see (29–31)].

In this paper we aim to outline how one Australian state has responded to the problem of youth radicalisation through the development of a CVE approach for at-risk and radicalised youth offenders. This paper draws on the existing literature and experiences of the authors in developing and implementing the Youth Justice New South Wales (NSW) CVE framework. A systematic literature review was not conducted. Rather, the authors completed a general review of the available academic and grey literature. The literature has contributed to the ways in which Youth Justice NSW has approached youth radicalisation and some of the challenges discussed in this paper. No interviews were conducted with staff from Youth Justice NSW or other Australian jurisdictions. Little is known about best-practise approaches for responding to youth radicalisation. This paper subsequently helps provide insights regarding the types of approaches that can be adopted.

This paper is organised as follows. First, we will discuss trends in youth radicalisation and the available literature regarding CVE approaches for youth. We will then outline the development of a practitioner-based model for the identification, assessment, and case management of at-risk and radicalised youth offenders. This is underpinned by consideration for the impact of psychosocial and developmental factors on youth vulnerability to exposure to and influence from extremism. This includes supplementing youth-specific CVE practitioner insights with consultation and capacity building support for frontline staff, as well as the implementation of a range of violent extremism risk assessment measures. This paper will close by discussing limitations in the field and future challenges. The Youth Justice NSW experience indicates that youth criminal justice settings can be designed to address the complex challenges posed by at-risk and radicalised youth.

## Youth Radicalisation in a Global and Australian Context

This section will position the presence of at-risk and radicalised Australian youth within a global climate of youth radicalisation to a variety of extremist groups and movements. Accounts of radicalisation have tended to imply that ideologically motivated violence is primarily an adult issue (32). This misnomer is supplemented by suggestions that the presence of youth in global matters of national security is mostly a modern occurrence (33). However, throughout history youth have been radicalised across a vast array of political, civil, social, ethnic, cultural, and religious contexts (34–36). This includes the presence of radicalised youth within the anti-colonial campaigns and partisan resistance movements that played out across the world in the early to-mid 1900's (35), youth engaged in the nationalist sentiment evoked within the initial iterations of the Irish Republican Army (37, 38), radical political rhetoric promoted across Zionist and Palestinian youth movements (35), and the attempted systematic indoctrination of a whole generation of youth within Hitler's Nazi Germany (32).

Youth were radicalised across revolutionary and liberation movements in the mid to late 20th century (3, 39–41). The global rise of far-right extremism is also particularly notable given that radicalised youth have a historical and contemporary presence across the complex landscape of white supremacist, neo-Nazi, fascist, nationalist, and socialist movements in North America and Europe (42–45). Youth have more recently been charged and convicted of right-wing inspired acts of violent extremism in the United Kingdom and United States (46–48).

Despite notable historical examples, there are few extremist groups or movements that has more effectively targeted, recruited, radicalised, and mobilised youth to violent extremism as the Islamic State and its affiliates (e.g., Boko Haram). One of the defining characteristics for which the Islamic State will long be remembered is the promotion of youth as part of the cornerstone of its organisation and self-proclaimed Caliphate (49). The relationship between youth and violent extremism was widely normalised under Islamic State rule. This entailed the indoctrination of future generations of Islamic State citizens and soldiers through propaganda and training camps (41, 49, 50) and manipulation of the education system and religious institutions (7, 51–53). Youth subsequently appeared alongside adults in Islamic State propaganda material and on the battlefield (52, 54, 55). This is perhaps best illustrated by the publicised images of a 4 year old British boy detonating a car bomb, resulting in the death of three Islamic State captives in January 2016 (41, 56).

The Australian Security Policy Institute noted that a small number of Australian youth, including those below the age of 18, were radicalised and mobilised to travel to join jihadist groups in the Middle East (57). Some of these Australian youth were recorded engaged in fighting in areas of conflict (58). Some appeared in online propaganda making threats against the West and others perpetrated suicide bomb attacks (57). The radicalisation of Australian youth to Sunni-Islamist ideology was highlighted in October 2015 by an Islamic State-inspired domestic terrorist attack that resulted in the shooting death of a NSW Police Force employee. The perpetrator, Farhad Khalil Mohammad Jabar, was an Australian youth aged 15 at the time.

## Responding to Radicalised Youth Offenders

This section will review global approaches for working with at-risk and radicalised youth offenders. The study of at-risk and radicalised youth is emerging as an area of academic interest (1, 27, 28). Our understanding of youth radicalisation is however “rudimentary” [(41), p. 165]. Cherney et al. added that youth radicalisation remains a relatively “unexplored area of study in Australia and internationally” [(14), p. 17]. These limitations remain enduring despite the incarceration of radicalised youth offenders. This includes prior estimates that in-excess of one million youth below the age of 18 were incarcerated globally in relation to matters of national security (59).

The role of incarceration as a response to violent extremism has long been depicted as a strategy that further contributes to the radicalisation of offenders, rather than one that rehabilitates (60). Academics have examined the role of custodial management regimes [e.g., (60–65)]. The provision of CVE-specific offender services targeted at rehabilitation and reintegration has also been considered [e.g., (66–71)]. Despite a notable increase in the literature, there remains few academic contributions that specifically explore radicalised youth offenders and the role of the youth criminal justice systems tasked with their supervision and rehabilitation (72).

Examples that do exist include Iganski et al. who reviewed multimodal programs delivered to violent extremist and hate crime youth offenders across a number of Western contexts (73). Their findings identified consistent themes across programs including pluralistic and educational therapeutic approaches, group work and the establishment of pro-social interactions, anger management interventions, and program components that address prejudice, bias, and remorse. The authors concluded that across different countries and jurisdictions “there is a clear need for shared learning” [(73), p. 7]. Dean et al. reviewed the implementation of the Healthy Identity Intervention (74). The program is designed to be delivered to convicted extremists in England and Wales. It was piloted in one young offender institution and addresses factors including identity, values, ideology, cognitive rigidity, and motivation for offending. It was concluded that the program is “useful for addressing extremist offending” [(74), p. 3]. However, no specific reflections were provided regarding program delivery in youth criminal justice settings.

Capano and Molenkamp explored the operational challenges and multifaceted CVE-specific functions implemented across two separate youth justice institutions in Germany (75). Youth violent extremist offenders were dispersed across therapeutic units. Staff were provided with extremism-specific training. Violent extremism risk assessment tools were also implemented. The authors noted that the approaches adopted to work with radicalised youth offenders were consistent with general criminogenic frameworks. This included a rehabilitative focus through developmental considerations, psychosocial skill acquisition and support, educational opportunities, vocational training, leisure, and the promotion of pro-social interactions with other detainees. Capano and Molenkamp concluded that there is a need for more “experience, expertise, and further research” [(75), p. 11].

Rahim conducted a case study of a young male convicted and incarcerated for a terrorism offence in the United States (76). He examined how the process of deradicalization was promoted in youth criminal justice settings through pluralistic and inclusive approaches to custodial supervision and case management. Rahim also reflected upon the absence of rehabilitative opportunities following transfer to adult custody (76). These findings are highlighted by the International Centre for Counter-Terrorism and Global Centre on Cooperative Security who in a joint-paper cautioned that careful planning and continuity of care must be maintained for offenders transitioning from youth to adult correctional systems (72). Alonso and Delgado also examined the incarceration of youth convicted of membership of a terrorist organisation in Spain (77). They suggested that psychological and educational intervention approaches were inhibited by the complexity of the personality profiles of radicalised youth offenders. They also noted that social reintegration was unable to be adequately addressed in a number of cases due to some youth terrorism offenders being expelled from Spain post-release.

## The Youth Justice New South Wales Experience

This section will review the practitioner-based approach adopted by Youth Justice NSW for working with at-risk and radicalised youth offenders. United Nations principles strictly govern frameworks in which many youth justice systems function. This is detailed in guidelines including the United Nations Rules for the Protection of Juveniles Deprived of their Liberty (78), and the United Nations Convention on the Rights of the Child (79). These frameworks promote standards for youth detention, rehabilitation, and reintegration. However, there is a lack of empirically-grounded youth-specific evidence, or widespread and critical academic contributions regarding best practise for the supervision and case management of radicalised youth offenders. This limitation is notable since it is argued that CVE approaches should be tailored specifically for youth (15, 41, 80, 81).

Some academics have recommended that youth CVE approaches need to consider a variety of individual and social factors (1, 80, 82, 83). Borum and Patterson added that this should entail “a developmentally informed understanding” [(27), p. 1147]. This includes considering how youth radicalisation and diversion or disengagement is impacted by developmental processes and experiences. This entails factors such as self-esteem, identity, belonging, pluralism, grievances, trauma, empathy, social and family relationships, critical thinking, cognitive flexibility, ideological education, community engagement, and resilience (22, 28, 83, 84). The Australian Government concluded that these factors should be conceptualised through “individually tailored and multi-faceted intervention plans that account for risk factors and characteristics unique to individual young people” [(15), p. 6].

In 2018 in the Australian state of NSW, the youth criminal system established a CVE Unit. This was in response to a number of youth convicted for terrorism offences. Youth Justice NSW is a state government agency from the NSW Department of Communities and Justice. Youth Justice NSW is responsible for the care and supervision of youth below the age of 18 that are subjected to legal orders. This includes custodial and community-based supervision. Some offenders are also supervised in the youth criminal justice system until age 21. Youth offenders in NSW can be characterised by high rates of trauma, interpersonal violence, and pronounced socio-economic disadvantage when compared to the general population (85). These complex profiles require additional CVE assessment and case management considerations.

The Youth Justice NSW CVE Unit was described by the NSW State Government as the first youth-specific framework of its kind in Australia (see 23). It was designed to ensure that “young children who are exposed to radicalisation are given counselling and a pathway back to life in the community” [(23), p. 1]. CVE Unit responsibilities include comprehensive practitioner-based assessments of at-risk and radicalised youth offenders; consultation and capacity building to support staff to identify and case manage; and youth-specific CVE training. Youth CVE consultation and training opportunities are also made available to external government and non-government agencies. This includes child and adolescent mental health services, child protection agencies, CVE programs, and non-government organisations responsible for youth-care placements and complex case management frameworks.

The Youth Justice NSW CVE Unit has adopted a practitioner-based approach to preventing and countering violent extremism. This means that CVE practitioners work directly with at-risk and radicalised youth offenders, their families, and multidisciplinary frontline staff. This dynamic and systemic approach is designed to ensure that relevant youth offenders remain central to CVE processes targeted at effective identification, diversion, and disengagement outcomes. The presence of a CVE function aligns with emerging approaches in adult criminal justice settings internationally. A variety of jurisdictions have established specialised staff and units to act as “a central hub” [(64), p. 26] to guide detection and management approaches for radicalised offenders. It needs to be emphasised that the Youth Justice NSW CVE Unit does not have a security, intelligence, or law enforcement remit. It instead represents a practitioner-based framework designed to support relevant youth offenders and staff across custodial and community supervision environments in NSW.

It is noted that a separate CVE approach has been tailored for radicalised inmates incarcerated in adult correctional settings in NSW [see (70, 86)]. The adult approach also adopts a case management framework (70, 86). This includes pronounced consideration for the role of ideology, grievances, and extremist networks (70, 87). In contrast, Youth Justice NSW CVE policy and practises are designed to promote early-identification, diversion, and disengagement opportunities across youth community supervision and custodial environments. The Youth Justice NSW CVE Unit approaches youth radicalisation through the lens of vulnerability, where a variety of psychosocial and developmental factors promotes the risk of a small cohort of youth offenders being exposed to, influenced by, and engaged in extremism. The Youth Justice NSW approach is in-line with non-punitive and systemic youth criminal justice frameworks where developmental considerations underpin client-centred approaches for youth engagement, assessment, and intervention (88, 89).

Youth Justice NSW has adopted a dispersal model for radicalised youth offenders in custody. This includes youth charged and convicted for terrorism offences. Radicalised youth offenders are housed within the mainstream detainee population. This dispersal approach is supported by research which notes that there is no evidence that radicalised youth offenders should be isolated or segregated from other detainees in custody (90). This integrated approach to custodial placement is also grounded in United Nations frameworks for youth detention. The United Nations provides that all youth detainees are entitled to a proportionate custodial balance between security arrangements and opportunities to engage in pro-social and approved programs, activities, and interventions (78, 79).

However, the dispersal approach in custody is not without its challenges due to the risk of radicalised offenders influencing others. This risk may be pronounced for youth offenders given the psychological vulnerability, search for identity, desire for belonging, and susceptibility to peer influence that characterises child and adolescent development (33, 72, 91, 92). These factors may be exacerbated for some youth offenders due to psychosocial deficits, disadvantaged backgrounds, anti-social patterns of thought, and limitations in the presence of protective factors. Youth Justice NSW has subsequently introduced operational measures to reduce the risk of radicalised youth offenders influencing other detainees. This includes additional monitoring provisions, limiting the availability of some programs and activities such as work details with limited supervision, and increased screening of placements, visits, phone calls, and associations. These operational measures are designed to supplement work conducted by the CVE Unit. Radicalised youth offenders are only subjected to additional CVE supervision and monitoring requirements in the community if ordered by the NSW Children's Court or subjected to a control order regime by the Australian Federal Police.

### Early Identification and Referral

The early identification and referral of at-risk and radicalised youth is viewed as key to the effectiveness of CVE approaches (15, 22). However, concerns and limitations have been expressed regarding CVE services engaging at-risk youth due to potential or perceived vulnerability. The risk of stigmatisation and labelling through involvement with CVE services has been identified (22). For example, some academics have criticised the United Kingdom's Prevent Programme for promoting the stigmatisation of youth through mandatory reporting requirements and the prescriptive nature of referrals (16, 93).

The capacity of frontline staff to identify risk factors whilst accounting for youth vulnerability and development has also been questioned in the Netherlands (17). This highlights challenges for early identification given that normal developmental experiences can increase vulnerability to youth being exposed to and influenced by extremism (1, 27). Examples include processes of identity formation, ideological exploration, a search for meaning and significance, and a desire for security and belonging. Youth rebellion and frustration may also be mistaken for commitment to extremism (22). Early identification approaches are complicated further given that vulnerability to radicalisation is argued to be context dependent and subsequently varies across different countries (94, 95).

The Youth Justice NSW experience indicates that the at-risk space provides opportunities for youth to be engaged in early intervention, diversionary, and preventative CVE approaches. The benefit of this is to ensure pathways and trajectories of radicalisation do not become pronounced, entrenched, and lead to a commitment to engage in ideologically motivated violence. Youth Justice NSW addresses the at-risk space in relation to youth who have displayed beliefs, behaviours, associations, and/or engaged in settings which indicate the early stages of exposure to and exploration of extremism. For example, this can involve youth offenders that possess violent extremist materials or those that have made comments or statements of a violent extremist nature. This may occur in the absence of clear evidence regarding their level of ideological commitment.

One key priority of Youth Justice NSW is to minimise the risk of inappropriate referrals, which can have the unintended consequences of stigmatising youth. This process involves consultation opportunities and case conferencing arrangements with referring staff and agencies. This includes staff reviewing the reported concerns, patterns of behaviour, and relevant vulnerabilities of those youth offenders that are brought to the attention of the CVE Unit. This process is often complicated by the psychosocial instability and pre-existing action orientation to violence that characterises youth offenders in NSW (85). The CVE Unit accepts referrals from frontline youth criminal justice staff, external government agencies, law enforcement, as well as non-government agencies that work with youth. The referral screening process entails the CVE Unit accessing and collating available information. This comprises of youth criminal justice file information as well as holdings from child protection, education, other CVE programs and services, and law enforcement agencies. Information is also shared with mental health services through agency agreements for the purpose of case management. However, in some instances, client consent is required. Information sharing arrangements are designed to promote cross-agency collaboration, robust screening provisions, and careful consideration of each individual referral.

This flexible and integrated screening approach is also designed to identify and redirect unsuitable referrals. This may include facilitating contact with mental health services, religious and cultural mentors, or support for local staff to understand and address reported issues of concern. This can occur without youth offenders at-risk of radicalisation having direct contact with the CVE Unit. This consultative process allows for referring staff and agencies to be educated and further supported regarding risk factors and approaches for identifying and working with at-risk and radicalised youth. However, a small number of youth offenders may benefit from comprehensive assessment. Before a referral is formally actioned, the CVE Unit provides a case presentation to what is termed the High Risk Young Offender Review Panel. The panel includes oversight from Executive-level Management in Youth Justice NSW and independent agencies including child protection, and child and adolescent forensic mental health services. The panel has sole authority for approving the CVE Unit to proceed with client-engagement and an offer to engage in a voluntary assessment.

### Assessment

One part of the Youth Justice NSW CVE framework is the completion of specialist comprehensive assessments. These assessments guide the development and implementation of individually-tailored case management plans. These plans are designed to address developmental and psychosocial vulnerabilities, criminogenic risks, and protective or risk mitigating factors. CVE considerations form only one part of the assessment process, which accords with arguments made in the literature that youth CVE considerations should be captured within a broader suite of assistance (28). CVE Unit assessments are conducted by specialist and trained youth CVE practitioners including psychologists and caseworkers. Participation in an assessment is voluntary and informed consent is required. If consent is not provided, a file review summary may be conducted, and general case management recommendations made. Attempts are made to re-engage youth offenders that decline consent to participate. This occurs in consultation with staff involved in the young person's supervision after a period of time has elapsed. CVE Unit assessments can cover youth offenders considered at-risk of radicalisation and those that have already radicalised to violent extremism. This includes youth convicted for terrorism offences.

A broad range of file information is generally available for review prior to initiating an assessment. Like many youth justice settings, the NSW framework adopts a client-centred approach (88). File information generally entails a range of information sources from identified agencies and will be supplemented by information collected from family members and key support people. Further information regarding ideological commitment, identity, knowledge, and practises is also sought as part of the assessment process. This can involve for instance general information and insights garnered from internal pastoral care services who may have had contact with a referred youth, and comprehensive theological assessments provided by experts external to Youth Justice NSW.

CVE Unit assessments can entail an extended period of in-person clinical interviews conducted by psychologists. This can include for example clinical assessments related to impulsivity, poor behavioural and emotional controls, immaturity, cognitive functioning, limitations in consequential thinking, reward seeking conduct, trauma, and susceptibility to peer and family influence. A range of youth-specific psychometric and criminogenic tools are also implemented to promote accurate assessments. These are chosen for implementation on a case to case basis. This includes standardised assessment measures that explore individual factors such as personality traits and characteristics, cognitive skills and capacities, trauma, anti-social attitudes, violence and delinquency, and protective factors. Examples of these available tools include the Wechsler Intelligence Scale for Children—Fifth Edition (WISC-V), Minnesota Multiphasic Personality Inventory—Adolescent (MMPI-A), Trauma Symptom Checklist for Children (TSCC), Structured Assessment of Violence Risk in Youth (SAVRY), and the Structured Assessment of Protective Factors for Violence Risk (SAPROF) tool. These assessments are designed to contribute to clinical case formulation, responsivity considerations, and recommendations regarding individually-tailored case management plans. Standardised psychometric and criminogenic tools are also utilised to assist in the interpretation and formulation of results elicited from more specific and targeted violent extremism risk assessment measures.

### Violent Extremism Risk Assessment

There are few pockets of the CVE landscape that appear to elicit more attention, commentary, and controversy as the implementation of violent extremism risk assessment tools. The small suite of available measures have been developed on the premise that whilst it may not be possible to provide a statistical prediction of violent extremism risk, “it may well be possible to construct a tool which will be able to assess the dangerousness of radical extremists” [(96), p. 5]. These tools have been promoted since it is generally agreed that criminogenic measures fail to account for factors specific to radicalisation and violent extremism (97–99).

The Youth Justice NSW CVE Unit has trailed the use of a number of tools for implementation with youth. This includes the RADAR tool (100), Terrorist Radicalization Assessment Protocol (TRAP-18) (101) and Building Resilience Against Violent Extremism (BRAVE) measure (102). The CVE Unit adopts the RADAR risk screening component to supplement information collected for consideration in the referral process. The TRAP-18 is used to supplement the assessment of youth displaying concerns of lone actor trajectories to violent extremism in the community. Our experience has found that the operational and investigative nature of the TRAP-18 appears to limit its utility for case management purposes for youth in custodial settings. The BRAVE provides a unique measure of protective and resilience factors regarding radicalisation and violent extremism. However, the BRAVE tool is normed for youth over the age of 18 only.

The main assessment tool adopted by Youth Justice NSW has been the Violent Extremism Risk Assessment – Version 2 Revised (VERA-2R) (103). The tool has been endorsed by the State and Federal Government for implementation within CVE programs and initiatives (12, 104). The VERA-2R is a specialised violent extremism risk assessment tool using the Structural Professional Judgement (SPJ) framework (103). The SPJ approach has been promoted for the assessment of radicalisation and violent extremism risk. Logan and Lloyd concluded that SPJ “offers the greatest potential for evidence-based, proportionate, transparent, and accountable practise” [(105), p. 145]. The SPJ approach has been adopted for the development and implementation of a number of youth-specific criminogenic assessment measures [see (106–108)]. This includes the Structured Assessment of Violence Risk in Youth (SAVRY) upon which the VERA-2R framework was partially modelled (103).

The VERA-2R is based on existing literature as well as consultations with law enforcement, intelligence, national security, and correctional professionals working in the field of violent extremism (103). Youth Justice NSW did contribute feedback to the authors of the VERA-2R regarding the applicability and definitions of some indicators following initial training of staff and implementation with youth. The VERA-2R consists of 34 items that are designed to capture contextual factors and risk indicators relevant to radicalisation and violent extremism. This occurs across a number of predetermined domains. The VERA-2R contains an additional 11 indicators that may be relevant to the risk of people being radicalised to engaged in an act of violent extremism. These additional indicators include consideration of prior youth criminal activities, issues in educational settings, problematic personal histories, and personality and mental health pathology. The authors of the VERA-2R argue that the tool is relevant for use across both gender and the life-course stages, including implementation with youth (103). It should be emphasised that the VERA is used by Youth Justice NSW to supplement CVE assessments and the development of case management plans. It is not a tool that the CVE Unit relies upon exclusively because of the explicit strategy to adopt a wide-ranging assessment process that can account for numerous vulnerabilities and needs.

The benefit of the VERA tool is that it is used to provide insights specific to radicalisation and violent extremism. It is not adopted to directly assess youth-specific developmental or psychosocial factors and hence it is used to compliment the assessment process and resulting case formulations. VERA-related data is elicited from file information, clinical interviews, and standardised psychometric and criminogenic measures. The CVE Unit facilitates and triangulates the scoring of the VERA-2R through a multidisciplinary panel of certified users and youth practitioners and professionals. This is designed to encourage robust discussions regarding individual indicators and to ensure consistency in its application.

The implementation of the VERA-2R with youth has raised some challenges for Youth Justice NSW. The authors of the VERA-2R argue that the tool is based on the current state of knowledge regarding radicalisation and violent extremism (103). However, the study of at-risk and radicalised youth is an emerging area of academic interest that remains relatively unexplored (1, 14, 28). There is a lack of empirical data or critical review regarding the utility of the VERA-2R for implementation with youth. It has also been argued that tools like the VERA-2R fail to adequately account for the developmental, cognitive, psychological, and emotional immaturity of youth (13, 33, 90).

Based on the Youth Justice NSW experience, some of these concerns may not be as pronounced in practise as initially thought. The vast and variable nature of radicalisation and violent extremist behaviour has led to the adoption of an equally broad set of risk indicators by tools like the VERA-2R. Such a wide-ranging SPJ framework may therefore promote the capacity of skilled and experienced assessors to incorporate individual and environmental factors relevant to youth development and vulnerability. An example includes accounting for the role of emotional dysregulation in youth that have made comments or threats of a violent extremist nature. It is important to note that developmental vulnerabilities amongst youth can both promote and mitigate risk. This can include for example processes of identity exploration and formation. This could leave some youth susceptible to influence from extreme associates whilst at the same time promoting opportunities to support engagement and exploration of pro-social activities and environments.

The Youth Justice NSW experience indicates that violent extremism risk assessment measures should incorporate and consider factors related to psychosocial and developmental vulnerability. It is also important that violent extremist assessments avoid the simple coding and ranking of individual indicators on risk assessment measures. In practise, this involves formulating risk scenarios and case management plans that cut across individually-specific characteristics, vulnerabilities, and environmental factors. This can include for example mapping risk scenarios for a socially isolated youth offender that has established associations with extremist peers in the context of suffering from a pervasive developmental disorder that promotes social isolation and a desire for belonging, emotional dysregulation, poor behavioural controls, impulsive violence, and a pronounced susceptibility to influence. This will differ from the development of risk formulation and scenarios for a youth offender who suffers from no developmental or psychological disorder, but has radicalised to violent extremism in the context of extremist family networks, perceived community grievances, and an elongated process of ideological indoctrination via family influence and social media. Hence the assessment framework adopted by Youth Justice NSW is driven by a holistic approach which ensures the process is not reliant upon a single assessment measure or data source.

### Case Management

It is beyond the scope of this paper to provide an empirical assessment of the effectiveness of CVE case management processes implemented by the Youth Justice NSW CVE Unit. What can be stated is that the assessment process outlined above guides the development of individually-tailored case management plans that make use of pre-existing resources within and external to Youth Justice NSW. This includes psychologists, caseworkers, youth officers, mentors, teachers, program facilitators, and chaplaincy services. Individual counselling and group-based programs are implemented, with a focus on psychological and educational or vocational needs, sports, leisure, life-skills, future planning, family support, and community engagement. This widespread allocation of resources is available to all youth offenders supervised within the youth criminal justice system in NSW.

The CVE Unit adopts a consultation and supervisory approach to the implementation of case management plans for at-risk and radicalised youth. Pre-existing multidisciplinary practitioners and programs are supported by the CVE Unit to ensure that relevant risks of radicalisation and violent extremism are incorporated and addressed. The CVE Unit monitors and amends case management plans in-line with updated assessments. This includes revised violent extremism risk assessment profiles. Regular follow-up is recommended for at-risk youth (109). This is important given it has been suggested in the literature that CVE outcomes may be time limited in the absence of ongoing support (22). This is reinforced by increasing commentary regarding the assessment and supervision of enduring or residual risk (104, 110).

In addition to support and consultation, the CVE Unit has developed a number of youth-CVE training packages. Their content is designed to increase staff capacity to understand the relevance of youth to extreme groups and movements, global trends and challenges, and to guide the identification and reporting of indicators that should underpin referrals and consultation opportunities. Training content is also designed to review approaches for working with at-risk and radicalised youth, and to promote a developmentally informed understanding of the process of youth radicalisation. For example, there is a focus on understanding the impact of vulnerability in youth that are exposed to and influenced by extremism. Youth-specific training content was required given it was identified that exiting government training packages tended to focus almost exclusively on trends and theories regarding radicalised adults. It was identified that these standardised packages failed to account for the nuances of at-risk and radicalised youth, the unique contexts in which they engage services, and the needs of youth practitioners and agencies. Capacity building opportunities have been recommended as part of youth CVE frameworks (22).

## Future Challenges

This section will address future challenges in relation to working with at-risk and radicalised youth. There are a number of notable youth-specific CVE challenges that confront policy-makers and practitioners. The continued evolution of policy and practise is impacted by the shifting CVE climate currently being observed across much of the Western world. There is little evidence to suggest that the threat posed by youth radicalised to Sunni-Islamist ideology will significantly subside in the foreseeable future. However, additional policy and practise challenges may be posed by the presence of at-risk and radicalised youth returning from conflict zones, in new or emerging extreme ideologies and movements, and within extreme far-right spaces.

The evolving nature of Western CVE landscapes has been highlighted in the United Kingdom where concerns in relation to far-right extremism now constitute the highest proportion of referrals to the Prevent Programme (5, 111). More than half of these referrals are for youth under the age of 20. More recently, counter-terrorism police disclosed that approximately three-quarters of British minors arrested for terrorism offences in the year to April 2021, were linked to far-right extremism (112). Concerns regarding youth radicalisation to far-right extremism have also recently been expressed by Australia's chief law enforcement and intelligence agencies (18, 19). This has been reflected in the emergence of extreme far-right referrals to the Youth Justice NSW CVE Unit. Preliminary observations indicate the presence of a varied range of right-wing-inspired narratives which are also currently mirrored more broadly across Australia's emerging extreme far-right landscape (113, 114). There remains much to learn in relation to best-practises approaches to responding to youth engaged in far-right extremism.

The impact of social isolation, uncertainty, and increased online activities as a result of the Covid-19 pandemic may also present future challenges. The pandemic has coincided with a growth in online extremist content (115). The degree to which youth have been exposed to and influenced by anti-system, grievance-based, and conspiratorial rhetoric related to Covid-19 remains to be seen. Emerging trends and challenges across Australia's domestic CVE landscape may therefore promote the need to continue to adjust CVE identification, reporting, assessment, case management, and training approaches.

The Youth Justice NSW experience also indicates that there is scope for further collaboration and consultation between youth CVE frameworks and mental health practitioner including those represented on fixated threat assessment committees. This should entail consideration of youth who present with a complex mix of threats and fixations, mental health and/or personality pathology, developmental vulnerability, and interest in extreme rhetoric and materials. Examples include youth that have consumed extremist content and made threats towards public institutions including schools. Increased collaboration may promote more effective screening processes, and the allocation of resources and expertise to ensure complex youth risks and needs are adequately assessed and addressed.

Youth Justice NSW faces challenges regarding the transfer of radicalised youth offenders to adult criminal justice systems. This includes youth convicted for terrorism offences. The challenges surrounding these transitions are notable given the fact that the rehabilitative, individualised, client-centred, and systemic approaches promoted across youth criminal justice settings may not be easily transferrable to the inherently larger, security-driven, and potentially punitive custodial regimes that await radicalised offenders in some adult correctional jurisdictions (62). The complexity of these transfers was captured by the International Centre for Counter-Terrorism and Global Centre on Cooperative Security who noted that, “the transition from a juvenile system to an adult prison can have far-reaching implications for the transferred youth” [(90), p. 10)].

The notion of youth-specific nuances and the evolution of CVE practise is also relevant for the ongoing implementation of violent extremism risk assessment measures. In the context of NSW, it appears the VERA-2R possesses value for use with at-risk and radicalised youth offenders in an Australian context. However, empirical evidence and comprehensive evaluation are needed to gauge how the VERA-2R can be updated to increase its practical utility and validity for use with at-risk and radicalised youth. Similar consideration should be given to screening tools additional risk assessment or case management measures that supplement early identification and diversionary processes. Recent court cases in Australia relating to the ongoing detention of convicted terrorism offenders have focused on the validity and accuracy of VERA-2R assessments to be used to assess current and on-going risk [see for example (116)]. The context of the NSW Children's Court for the use and application of the VERA-2R remains relatively unexplored. The Youth Justice NSW CVE Unit has appeared in the NSW Children's Court in relation to CVE matters. However, thus far the VERA-2R has remained unchallenged.

Youth-specific professional development and supervision forums also appear to be lacking for CVE practitioners across Australia's domestic CVE landscape. Youth Justice NSW is in the early stages of contributing to the establishment of a number of forums to address this. These will provide opportunities for youth CVE practitioners to share insights, experiences, case reviews, and learnings. A CVE-specific evaluation plan has also been established for the Youth Justice NSW CVE Unit. This evaluation will canvass the effectiveness of CVE engagement processes, draw on stakeholder consultations, and identify opportunities to increase capacity and training.

## Conclusion

Youth radicalisation represents an ongoing challenge. The Youth Justice NSW approach understands youth radicalisation as the result of a mix of psychosocial and developmental vulnerabilities, and risk factors. However, the establishment of best practise approaches to assess, prevent, and counter violent extremism in youth still remains relatively unexplored.

This paper has outlined how one Australian state has addressed youth radicalisation through the implementation of a practitioner-based framework across the youth criminal justice system. This includes early identification and diversionary opportunities and the implementation of various risk assessment measures. Any extremist risk assessment measure should be used in conjunction with collateral information, clinical interviews conducted by experienced CVE practitioners, and results elicited from standardised psychometric and criminogenic assessment tools that account for youth-specific needs including developmental vulnerability.

The authors argue that youth criminal justice settings promote unique opportunities to prevent and counter violent extremism in youth. This is due to widespread resourcing of multifaceted and multidisciplinary practitioners and programs. These resources can be supplemented by youth-specific CVE expertise. This should occur in the form of specialised CVE practitioners that conduct assessments and provide CVE consultation, support, case conferencing, capacity building, and training opportunities for youth staff and agencies. At-risk and radicalised youth pose challenges for the practitioners and agencies tasked with their assessment and case management. At-risk and radicalised youth also represent a demographic of great potential for behavioural change.

## Data Availability Statement

The original contributions presented in the study are included in the article/supplementary material, further inquiries can be directed to the corresponding author/s.

## Author Contributions

SB and JM conceived and designed the manuscript. SB wrote the manuscript. Both authors contributed to reviewing the manuscript.

## Conflict of Interest

The authors declare that the research was conducted in the absence of any commercial or financial relationships that could be construed as a potential conflict of interest.

## Publisher's Note

All claims expressed in this article are solely those of the authors and do not necessarily represent those of their affiliated organizations, or those of the publisher, the editors and the reviewers. Any product that may be evaluated in this article, or claim that may be made by its manufacturer, is not guaranteed or endorsed by the publisher.
